# An Accurate Electro-Thermal Coupling Model of a GaAs HBT Device under Floating Heat Source Disturbances

**DOI:** 10.3390/mi14122236

**Published:** 2023-12-13

**Authors:** Xiaohong Sun, Yijun Yang, Chaoran Zhang, Xiaodong Zhang, Ting Tian

**Affiliations:** 1 School of Electronic and Information Engineering, Suzhou University of Science and Technology, Suzhou 215009, China; yangyij1998@163.com (Y.Y.); wushengzcr@126.com (C.Z.); waxzl@hotmail.com (X.Z.); 2 High-Frequency and High-Power Laboratory, Southeast University, Suzhou 215123, China; tianting@innotion.com.cn

**Keywords:** GaAs HBT, self-heating effect, heat source, electro-thermal coupling

## Abstract

Taking into consideration the inaccurate temperature predictions in traditional thermal models of power devices, we undertook a study on the temperature rise characteristics of heterojunction bipolar transistors (HBTs) with a two-dimensional cross-sectional structure including a sub-collector region. We developed a current-adjusted polynomial electro-thermal coupling model based on investigating floating heat sources. This model was developed using precise simulation data acquired from SILVACO (Santa Clara, CA, USA). Additionally, we utilized COMSOL software (version 5.6) to simulate the temperature distribution within parallel power cells, examining further impacts resulting from thermal coupling. The research findings indicate that the rise in current induces modifications in the local carrier concentration, thereby prompting variations in the local electric field, including changes in the heat source’s peak location and intensity. The device’s peak temperature exhibits a non-linear trend regulated by the current, revealing an error margin of less than 1.5% in the proposed current-corrected model. At higher current levels, the drift of the heat source leads to an increase in the heat dissipation path and reduces the coupling strength between parallel devices. Experiments were performed on 64 GaAs (gallium arsenide) HBT-based power cells using a QFI infrared imaging system. Compared to the traditional temperature calculation model, the proposed model increased the accuracy by 6.84%, allowing for more precise predictions of transistor peak temperatures in high-power applications.

## 1. Introduction

Gallium arsenide (GaAs) heterojunction bipolar transistors (HBTs) stand out as prominent components in Monolithic Microwave Integrated Circuit (MMIC) power amplifier design, primarily owing to their remarkable attributes, including a high power density and elevated electron mobility. In the normal operation of a power amplifier, due to its limited efficiency, only a portion of the power supply energy is converted into RF output signals, while the remaining energy is transformed into power consumption. The dissipation of power generates heat in the transistor, leading to an increase in its temperature. This challenge is compounded by the limited thermal conductivity characteristics inherent in GaAs, intensifying the extent of temperature elevation. Consequently, this induces changes in the physical performance of semiconductor devices, compromising the linearity of the power amplifier and diminishing the output power. This, in turn, limits the power handling capability of RF power amplifiers at high frequencies [1,2]. As the landscape of wireless communication evolves towards long-distance, ultra-high-speed transmission, an increasing demand for RF power amplifiers characterized by a smaller size and an increased output power has arisen. The continuous increase in power density exacerbates the thermal effects in power amplifiers, resulting in more noticeable temperature rises in transistors and further deterioration of output characteristics such as efficiency, gain, and linearity. Thermal optimization techniques play a crucial role in enhancing the performance of RF power amplifiers. The exploration of thermal management optimization [3,4,5] in RF power amplifiers has become a research hotspot, and is expected to strongly drive and promote further advancements in RF power amplifier technology. In order to improve the effectiveness of thermal management and design RF devices with superior performance, the development and utilization of accurate electro-thermal coupling models [6] are imperative. These models play a crucial role in accurately predicting the temperature distribution, thereby contributing to the effective enhancement of thermal management strategies in RF power amplifiers.

Traditional electro-thermal studies often treat active devices as uniform rectangular heat sources fixed on the substrate surface, with the value depending on DC power dissipation. Typically, these studies involve numerical solution of the Laplace equation to calculate temperatures. However, this approach can be computationally intensive and dependent on the performance of the computer hardware. Reference [7], considering the device’s geometry and the shape of isotherms based on heat conduction simulations, deduced the thermal conductivity angles to analytically estimate the thermal resistance for temperature evaluation. Reference [8] employed Fourier’s law to consider thermal coupling and self-heating in multi-finger GaAs HBTs, deriving analytical expressions for the junction temperature in practical power unit structures. To provide a more precise characterization of the temperature increase, recent research has focused on the physical processes of heat source generation within the devices. References [9,10,11,12] suggested the utilization of Sentaurus simulation software (2018) with Monte Carlo algorithms to conduct precise thermal simulations considering alterations in lattice vibrations in devices such as HEMTs. In the specific context of GaAs HBT devices, reference [13] conducted an in-depth analysis of the thermal phenomena within the two-dimensional structure of the device. Notably, the study emphasized the impact of the sub-collector layer on the distribution of heat sources. This investigation provides invaluable insights for more accurate modeling of temperature rises in GaAs HBT devices, illuminating the critical factors that influence heat distribution within the device structure. As the electric field drifts, such nuanced processes in thermal modeling are expected to contribute significantly to the advancement of electro-thermal analyses of semiconductor devices.

Based on precise simulations of two-dimensional single-transistor devices, a self-heating nonlinear model is proposed, accounting for the influence of a floating heat source disturbance. Unlike traditional models, which consider only power dissipation as a variable, this model incorporates variations in electric field distribution characteristics under high-current conditions. SILVACO simulation data from a single transistor are fitted to a nonlinear temperature analytical model. This model demonstrates high conformity with actual simulation results across a wide range of currents, with the prediction errors controlled to within 1.5%. The study further explores the dynamic nature of self-heating effects and the inter-thermal coupling strength in multi-transistor configurations, as influenced by the drift of the heat source center. To validate the proposed model in a practical setting, temperature testing was conducted on a power cell comprising 64 transistors by employing a QFI infrared imager. The results indicate that, particularly under high-current conditions, the proposed model outperforms traditional power dissipation models, yielding a noteworthy reduction in prediction error of 6.84%. This innovative self-heating nonlinear model not only refines temperature predictions but also introduces a nuanced perspective by considering electric field distribution characteristics. The reduction in prediction error underlines the model’s validity for capturing the complexities associated with high-current conditions, showing its potential to enhance the accuracy of thermal analyses of transistor devices.

## 2. Establishment of Theoretical Models

The simulation in this study was conducted using SILVACO Technology Computer-Aided Design (TCAD). SILVACO TCAD (2018) is a powerful software tool designed to predict the electrical behavior of specified semiconductor structures, offering insights into the internal physical mechanisms governing device operation. In our specific investigation, the semiconductor device was modeled within the ATLAS module of the SILVACO software (2018) in detail. The ATLAS module employs a numerical solution approach to solve the equations governing the behavior of the semiconductor device. This is achieved by calculating the values of unknowns on a mesh of points distributed within the device. To enhance the computational efficiency and convergence, a mixed technique combining BLOCK and NEWTON methods was employed as the calculation method. Both BLOCK and NEWTON methods are explicitly defined and implemented within the ATLAS module, contributing to an improved speed and convergence of the simulation process.

### 2.1. Single-Transistor Electro-Thermal Phenomenon

**Figure 1:**
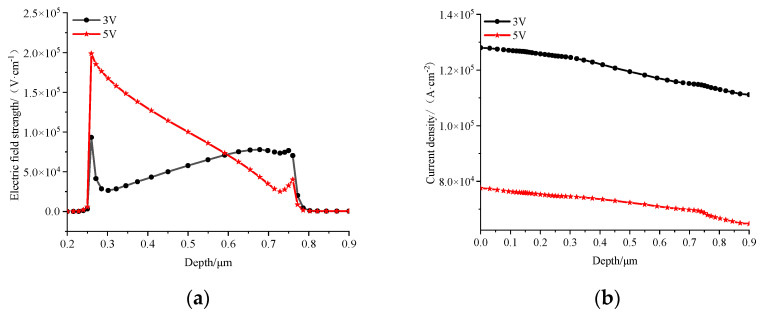
Biases of 3 V and 5 V with the same power consumption: (**a**) electric field distribution; (**b**) current density distribution.

**Figure 2:**
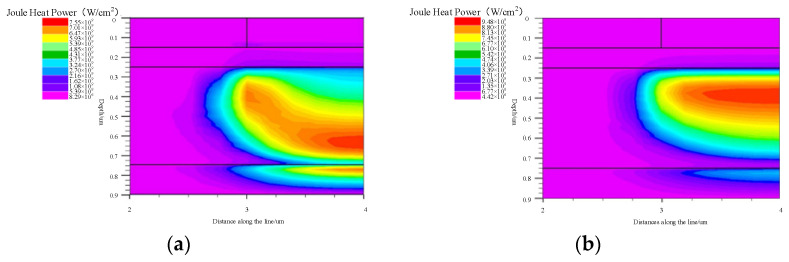
Joule thermal power distribution under the same power consumption: (**a**) 3 V; (**b**) 5 V.

This passage outlines a simulation process focused on the classic two-dimensional GaAs HBT structure with a current gain of 60 with normal forward bias. The key parameters for this simulation, including dimensions and doping concentrations, adhere to the specifications detailed in reference [14]. The structural configuration of the transistor is divided into four distinct regions, ordered from top to bottom: an emitter region, a base region, a collector region, and a sub-collector region. A crucial aspect of the simulation setup involves managing the thermal conditions of the sub-collector region’s base. This region is thermally connected to a constant-temperature environment via a thermal resistance, introducing a controlled thermal boundary condition. Simultaneously, the surface conditions are set as adiabatic, signifying no heat exchange with the surroundings at the external surface of the structure. To ensure the accuracy of the simulation, a comprehensive set of factors is taken into consideration. These factors encompass the effects of carrier generation–recombination, modeled through the Shockley–Read–Hall (SRH) model, on minority carrier lifetimes. Additionally, the simulation accounts for the impacts of electric fields and concentration on carrier mobility, as well as the speed saturation effects observed at high fields. The physical process is then simulated by solving Poisson’s equation, the carrier continuity equation, the energy balance equation, and the drift diffusion equation, along with lattice heating and heat flow equations. This set of equations encompasses one Poisson equation, describing the relationship between electric potential and position. There are also two continuity equations representing the relationship between electron and hole current densities and their time variations. The transport equation reflects the relationship between the electron current density and position, and the self-heating equation captures the interplay between heat generation and heat flow. In essence, these equations collectively constitute the governing equations that dictate the intricate interactions and behaviors within the GaAs HBT structure during the simulation process. By addressing the complex interplay of electrical and thermal factors, this simulation framework provides a robust foundation for understanding the device’s performance characteristics under diverse operating conditions. For this non-isothermal complex system, the physical models necessitate solving up to six coupled equations [14]. A mixed technique involving BLOCK and NEWTON is employed as the calculation method to enhance the speed and convergence. The BLOCK method solves some equations in a fully coupled manner while decoupling others. The NEWTON method addresses the entire system of unknowns in a fully coupled manner. In our simulation, BLOCK obtains a coupled solution for the Poisson and continuity equations using the Newton algorithm with a constant temperature, followed by a coupled solution for carrier energy balance and carrier continuity equations. If convergence remains elusive, the program switches to the fully coupled NEWTON method. Subsequently, it addresses the lattice heating equation in a decoupled manner. However, precisely because we have meticulously modeled the physical processes, even with the adoption of advanced computational methods, the calculation speed is relatively slow. Under this construction of the physical model, a single transistor calculation under appropriate bias conditions takes approximately 25 min. For multi-finger transistor calculations, the results often exhibit non-convergence. Figure 1a,b illustrates the distribution of the electric field and current density under 3 V and 5 V biases at the same power consumption, respectively. It is noticeable that, with a 3 V voltage bias, the electric field peak shifts from the surface to the sub-collector electrode due to the influence of a high current. The corresponding heat source distribution is presented in Figure 2. Under the 3 V condition in Figure 2a, the heat source is concentrated at the interface between the collector electrode and the sub-collector electrode. In the 5 V condition in Figure 2b, it is closer to the collector junction, exhibiting a higher peak and leading to an elevated junction temperature. It can be observed that even under the same power consumption, the variations in heat source size and location caused by two different biases are different. This leads to certain disturbances in the temperature rise compared to the predictions of traditional temperature models.

### 2.2. Physical Analysis

The increase in transistor temperature resulting from thermal dissipation fundamentally arises from alterations in the thermodynamic system, which comprises electrons, holes, and the semiconductor lattice. Under the influence of a strong electric field, charge carriers are accelerated and gain energy from the electric field, causing their average energy to be significantly higher than it is in equilibrium. During the scattering process, this extra energy is absorbed by the lattice, leading to an increase in temperature [15]. Therefore, changes in the heat source are related to variations in the internal electric field of the device. The magnitude of the local electric field can be described using Poisson’s equation; Poisson’s equation relates variations in the electrostatic potential to local charge densities by:

(1) $$\frac{dE}{dz}=\frac{q}{\varepsilon_{s}}(p-n+N_{D}-N_{A})$$

In this equation, *p* and *n* represent the concentrations of local holes and electrons, while *N_D_* and *N_A_* represent the ionized donor and acceptor impurity charge quantities and *ε_s_* is the relative dielectric constant. For the collector junction of an NPN-type HBT biased in reverse, the majority of carriers in the collector region is electrons, and the ionized impurities are mainly donors. Additionally, when the electric field intensity exceeds 10^3^ V/cm, carriers in the GaAs material move at saturation velocity; thus, the above equation can be simplified to:

(2) $$\frac{dE}{dz}=\frac{q}{\varepsilon_{s}}(-\frac{J_{C}}{qV_{sat}}+N_{collector})$$

When the current density is low, the impact of the current can be neglected, and the peak electric field mainly appears at the surface of the collector junction, where the slope of electric field variation with depth is relatively steep. As the current gradually increases, the slope decreases and tends towards zero and the peak electric field at the collector junction also decreases. When the current density continues to increase, due to the heavy doping of the sub-collector electrode, the depletion region cannot extend further into the sub-collector region. As a result, the peak electric field occurs at the boundary between the collector and sub-collector electrodes, and its value gradually increases. Neglecting the thermal generation rate caused by generation–recombination processes, according to the drift diffusion model theory, the steady-state thermal generation rate can be expressed as follows:

(3) H=J×E

The product of current density (*J*) and electric field strength (*E*) constitutes the primary component of Joule heating. The distribution of the current density under the two different biases shown in Figure 1b exhibits a similar decreasing trend. Therefore, the thermal power distribution characteristics are consistent with the electric field distribution, as shown in the trend depicted in Figure 2.

(4) $$C\frac{\partial T_{L}}{\partial t}=\nabla (k\nabla T_{L})+H$$

Giga adds the lattice heat flow equation of Equation (4) to the primary equations. *C* is the heat capacitance per unit volume, *k* is the thermal conductivity, *H* is the heat generation, and *T_L_* is the local lattice temperature. As a result, variations in the value and location of the heat source lead to changes in the lattice temperature.

### 2.3. Nonlinear Temperature Model Based on Current Correction

The previous analysis and simulation results indicate that as the current increases, the center of the heat source drifts [13], subsequently influencing the temperature rise. Therefore, a nonlinear electro-thermal coupling model with current correction is proposed, as depicted in the following equation:

(5) $$T=f(U,I)=T_{0}+k_{1}\cdot (U\cdot I)+k_{2}\cdot (U\cdot I^{2})$$

**Figure 3:**
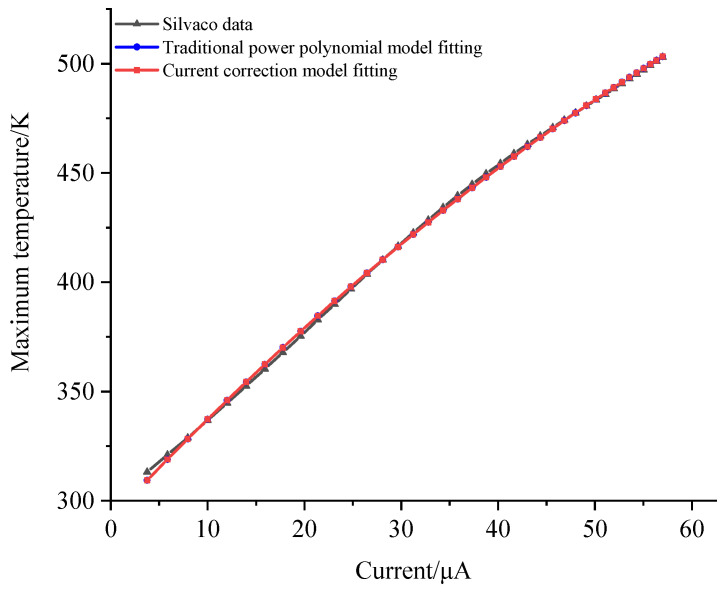
Fitted curve at a 4 V bias.

In this equation, *U* and *I* represent the collector bias voltage and current, respectively. *k*_1_ is a traditional thermal-resistance-like parameter, while *k*_2_ is a correction polynomial parameter that accounts for changes in the electric field distribution due to high current effects. Utilizing SILVACO’s precise calculations with a collector bias voltage of 4 V and a base current ranging from 9.4 × 10^−5^ A/μm to 1.4 × 10^−3^ A/μm, we obtained the desired temperature and collector current magnitude. Data fitting was performed using the MATLAB 2018 program, and the results are illustrated in Figure 3. The figure simultaneously compares the fitting effects of the traditional power polynomial model. The temperature rise can be represented as the first-order effect of power consumption (*p*), denoted as *R*_*th*1_, and also includes the correction of the second-order effect, denoted as *R*_*th*2_:

(6) $$T=f(p)=T_{0}+R_{th1}\cdot p+R_{th2}\cdot p^{2}$$

**Figure 4:**
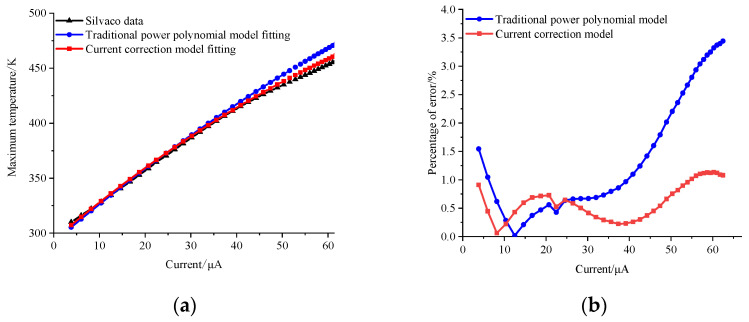
(**a**) Predicted temperature curve at a 3 V bias; (**b**) error distribution.

**Figure 5:**
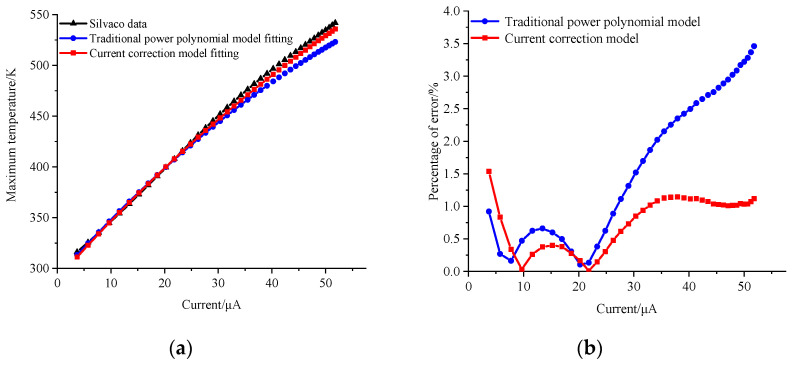
(**a**) Predicted temperature curve at a 5 V bias; (**b**) error distribution.

Figure 4 and Figure 5 offer a comparative analysis of temperature predictions for the transistor under 3 V and 5 V bias conditions, employing two distinct models. Notably, the traditional power polynomial model demonstrates a superior accuracy at low currents but exhibits a notable deviation from actual temperatures as the current increases. In contrast, the current-corrected model showcases a remarkable capacity to maintain prediction errors within a narrow margin of 1.5% across a broad spectrum of current magnitudes. Moreover, it achieves a substantial reduction in error from 3.5% to 1.1% in high current states. This underscores the current-corrected model’s efficacy in providing a more precise and reliable prediction performance, especially under conditions of elevated currents.

### 2.4. Temperature Distribution of Multi-Tube Parallel Units

In practical applications of HBT power amplifiers, multiple parallel power cells are commonly employed to achieve a high power output. For a multi-finger parallel structure, apart from the self-heating effects, the inter-thermal coupling effect modifies the distribution of the temperature field. The parallel configuration of multiple transistors displays a non-uniform distribution, resembling a bell curve, with the transistor situated in the middle of the layout experiencing the highest temperature.

**Figure 6:**
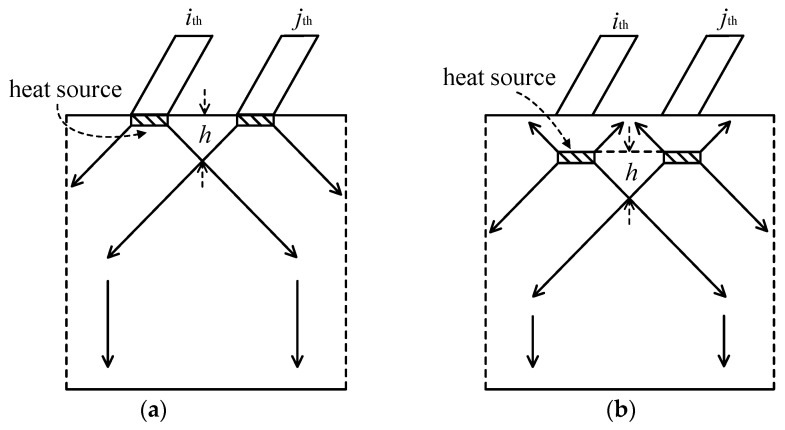
Thermal schematic of heat source drift. (**a**) initial position; (**b**) after drifting.

The drift of the thermal source introduces additional complexities to the temperature distribution due to self-heating and mutual heating. Figure 6a,b below illustrates the schematic diagram of thermal effects during heat source drift. In Figure 6a, the mutual heating coupling in a multi-finger parallel structure modifies the heat flow direction of individual fingers. Analyzing the variations in the isothermal lines reveals an approximate division into two parts: constant-angle transmission and vertical downward transmission. The transmission angle (thermal conduction angle) is governed by the structural parameters of the transistor (finger length, finger spacing, finger width) and substrate thickness. The figure illustrates the geometric approximation curves of the isothermal lines. When the heat source drifts toward the sub-collector region (Figure 6b), although the majority of the heat still flows towards the substrate, there is a portion of the heat flowing in the direction of the surface. It creates additional heat dissipation paths, consequently mitigating the temperature rise caused by self-heating. Regarding thermal coupling, the *i*-th finger, influenced by the adjacent *j*-th finger, exhibits an increased Δ*T_ij_*. Reference [8] provides an approximate value for the onset distance of thermal coupling, denoted as the size of *h*, determined by the substrate thickness and finger spacing. Consequently, the upward heat flow has not yet reached the point of thermal coupling, and the mutual heating resistance generated by the downward heat flow is further reduced. This reduction in mutual heating resistance disrupts the temperature rise, contributing to disturbances in the overall temperature distribution.

To further investigate temperature disturbances in parallel multi-finger power cells, computational simulations were conducted using COMSOL software. This can provide a steady-state temperature field analysis by establishing a three-dimensional model. The heat generated at the device induces a temperature difference between the heat source and other regions of the chip, as well as the surrounding medium. Following the principles of the second law of thermodynamics, heat is transferred between objects with varying temperatures, and in semiconductor devices, this is expressed through heat conduction. In contrast to SILVACO, which considers internal physical fields, here, each active region is treated as a uniform overall heat source. Material coefficients, such as the temperature-dependent thermal diffusivity, are selected to be consistent with those in SILVACO. Additionally, we make the following assumptions: (1) the substrate has a thickness of 100 μm, with a length and width both equal to 1000 μm, (2) the substrate surface is adiabatic, neglecting the heat dissipation effect of upper metal leads, and (3) the physical field only considers the heat conduction process, simulated and solved through a well-defined mesh partition. Here, the governing equation for the steady-state lattice heat diffusion is given as:

(7) ∇(k⋅∇T)=q

where *T* represents the steady state temperature, *k* represents the thermal conductivity (at room temperature, the thermal conductivity of GaAs is 46 W/m·K), and *q* represents the power generation per unit volume in the heat source.

**Figure 7:**
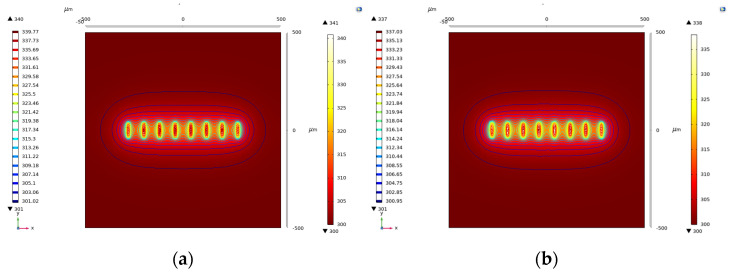
Thermal simulation results of the power cell in COMSOL. (**a**) before drifting; (**b**) after drifting.

In our simulations conducted with COMSOL, we represent the heat source as a uniformly distributed value and specifically examine its impact on the temperature rise when it experiences drift. The active device is simplified in our model as a rectangular structure with dimensions of 5 μm × 16 μm. The spacing between fingers within the power unit is set at 10 μm, and a DC power consumption of 1 W is applied to capture the thermal effects. The results of the temperature calculations are visually depicted in Figure 7a,b. Observations from the figures indicate that when the heat source is positioned on the surface, the maximum temperature rise is 40.96 K. However, when the heat source undergoes a downward drift of 1μm, the maximum temperature rise decreases to 37.96 K, representing a reduction of 3 K. This alteration corresponds to a disturbance of 7.9%. These findings highlight the sensitivity of the temperature distribution to the spatial location of the heat source, emphasizing the importance of considering such factors in the design and analysis of multi-finger power cells.

## 3. Experiment and Results Analysis

**Figure 8:**
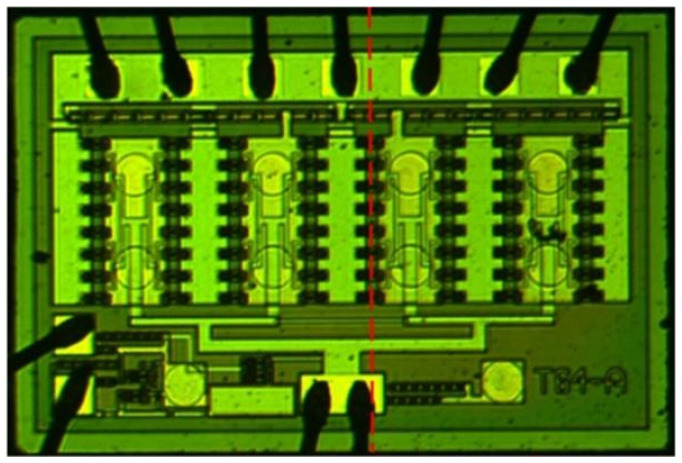
Transistor power cell for testing (Observed temperatures along the red route).

**Figure 9:**
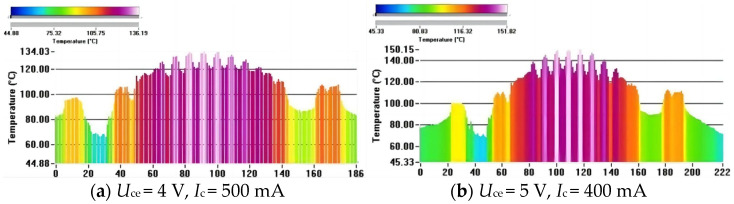
Temperature distribution at the dashed line.

In the utilization of a multi-finger power cell with a typical inter-finger spacing ranging from 5 to 10 μm, the thermal coupling strength exhibits variations as the heat source drifts, transitioning from the surface to the sub-collector region. To experimentally validate the model’s accuracy, a power cell designed for power amplifier applications was employed. This power cell comprises a uniformly distributed arrangement of 64 transistors organized in eight rows and eight columns. The active area of each transistor is 80 μm^2^, and the substrate thickness is 100 μm. After applying a DC power of 2 W to this power cell, the temperature distribution was observed along a cutline, as depicted in Figure 8. Figure 9 showcases the experimental results. Under a collector bias voltage of 4 V and a bias current of 500 mA, the highest temperature recorded was 136.19 °C. Conversely, at 5 V and 400 mA bias conditions, the highest temperature was 151.82 °C. This observation indicates that, at the same power consumption, transistors with a higher current experience a lower temperature.

**Figure 10:**
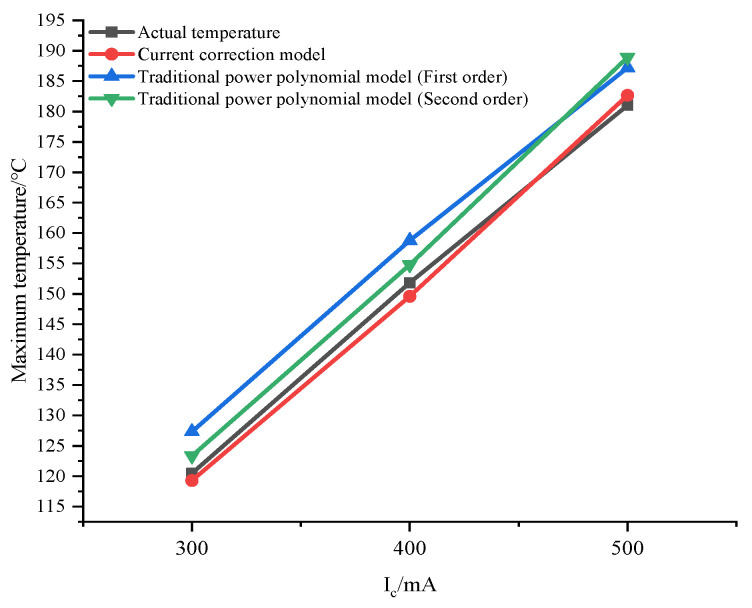
Comparison of the power polynomials and the current-corrected model (substrate temperature: 45 °C).

Under a bias voltage of 4 V, with a bias current varying in increments of 50 mA from 100 mA to 600 mA, the parameters for the current-corrected model and traditional power consumption model were computed based on voltage–current data. Subsequently, this allowed for the prediction of the maximum temperature of the device surface under the condition of 5 V. Examination of the data in Table 1 reveals that the current-corrected model exhibited an error of 1.5% compared to the measured temperature. In contrast, the errors associated with the first-order power polynomial and second-order power polynomial models reached a maximum of 8.34% and 4.35%, respectively. As can be seen from Figure 10, the prediction results of the first-order power polynomials deviate more from the actual data as a whole, and the results of the second-order power polynomials also deviate more at higher currents. The results of the current-corrected model are much closer to the actual temperature data and remain reliable at high currents. This discrepancy highlights an improvement in accuracy of 6.84% with the utilization of the current-corrected model. These findings underscore the enhanced predictive capabilities and precision offered by the newly introduced modified model in capturing temperature variations under varying bias conditions.

The parameters fitted by this model are crucially based on the fitting parameters obtained under a bias voltage of 4 V, ensuring highly accurate predictions for the temperature model under the conditions of 3 V and 5 V. Nevertheless, this implies a certain limitation, suggesting that significant deviations from this voltage condition may render the fitted parameters less accurate. Fortunately, the HBTs in the power amplifier design we employed typically operate with bias voltages that do not exceed 6 V. Furthermore, the introduction of corrective parameters to the traditional model enhances its accuracy significantly under high-current conditions. Conversely, at low currents, the impact of the corrective factors is minimal, resulting in an accuracy aligned with that of the traditional model.

## 4. Conclusions

This study investigated the establishment of a steady-state electro-thermal coupling model for GaAs HBTs. We proposed the inclusion of a current-corrected term to account for changes in the distribution of the internal electric field intensity and its impact on the temperature rise. In power amplifier applications, it was observed that as the electrical power dissipation increases, the peak temperature of the device also rises. However, under higher currents with the same power dissipation, the peak temperature is relatively low due to changes in the electric field distribution characteristics. In comparison to traditional nonlinear power polynomial models, the current-corrected model proposed in this paper can be employed to establish more accurate device models and guide circuit temperature compensation design. Furthermore, it provides a reference for the calculation of the depth and size of the heat source as well as the temperature distribution resulting from the secondary current distribution formed by the thermal coupling of multiple transistors under dynamic signal excitations. The temperature calculation model presented in this paper is specifically suitable for HBT devices featuring sub-collector regions, irrespective of the material employed. For alternative device types, it suffices to treat the correction factors in this model as zero.

## Figures and Tables

**Table 1:** Data comparison between the power consumption polynomial and the current-corrected model (substrate temperature: 45 °C).

*I*_c_/mA	*P*_diss_/W	Actual Temperature Rise/°C	Power Consumption Polynomial (First Order)/°C	Power Consumption Polynomial (Second Order)/°C	Current-Corrected Model/°C
300	1.5	120.47	130.52	123.33	119.28
400	2.0	151.82	158.76	154.78	149.58
500	2.5	181.02	187.20	188.90	182.65

## Data Availability

Data are contained within the article.
